# Impact of Delayed Admission to the Intensive Care Unit from the Emergency Department upon Sepsis Outcomes and Sepsis Protocol Compliance

**DOI:** 10.1155/2017/9616545

**Published:** 2017-03-12

**Authors:** Michael Agustin, Lori Lyn Price, Augustine Andoh-Duku, Peter LaCamera

**Affiliations:** ^1^Department of Pulmonary and Critical Care, St. Elizabeth's Medical Center, Tufts University, Boston, MA, USA; ^2^Guam Regional Medical City, Dededo, GU, USA; ^3^Clinical and Translational Science Institute, Tufts University, 35 Kneeland Street, Boston, MA, USA

## Abstract

*Rationale*. The impact of emergency department length of stay (EDLOS) upon sepsis outcomes needs clarification. We sought to better understand the relationship between EDLOS and both outcomes and protocol compliance in sepsis.* Methods*. We performed a retrospective observational study of septic patients admitted to the ICU from the ED between January 2012 and December 2015 in a single tertiary care teaching hospital. 287 patients with severe sepsis and septic shock were included. Study population was divided into patients with EDLOS < 6 hrs (early admission) versus ≥6 hours (delayed admission). We assessed the impact of EDLOS on hospital mortality, compliance with sepsis protocol, and resuscitation. Statistical significance was determined by chi-square test.* Results*. Of the 287 septic ED patients, 137 (47%) were admitted to the ICU in <6 hours. There was no significant in-hospital mortality difference between early and delayed admissions (*p* = 0.68). Both groups have similar compliance with the 3-hour protocol (*p* = 0.77). There was no significant difference in achieving optimal resuscitation within 12 hours (*p* = 0.35).* Conclusion*. We found that clinical outcomes were not significantly different between early and delayed ICU admissions. Additionally, EDLOS did not impact compliance with the sepsis protocol with the exception of repeat lactate draw.

## 1. Introduction 

Sepsis occurs in approximately 2% of all hospitalization in developed countries and may account for 20% of intensive care unit (ICU) admissions [1–3]. Septic shock carries high mortality rate and has been the leading cause of noncoronary ICU deaths [3, 4]. The Surviving Sepsis Campaign (SSC), launched in 2002, was aimed to reduce mortality from sepsis and septic shock worldwide by the use of sepsis bundles [5]. Studies have suggested that increased compliance with sepsis bundles is associated with reduction in sepsis mortality [4, 6].

The evolution of illness while the patient remains in the emergency department (ED) is also an important determinant of outcome. Unfortunately, many critically ill septic patients board in the ED for a prolonged period of time. Prolonged EDLOS, defined as an EDLOS more than 6 hours, was based on the Canadian Association of Emergency Physicians (CAEP) recommendation which was also used as a performance indicator to assess the quality of care in US ED [7–9]. In addition, an admitted patient who spends more than 8 hours in the ED from their time of arrival is an accepted measure of “access block” according to Australian College of Emergency Medicine (ACEM) [10, 11]. There have been conflicting studies regarding the effect of ICU transfer time with survival for patients with sepsis. Some studies showed increased risk of mortality and hospital stay when ICU admission is delayed [12–14]. In addition, delayed admission consumes greater resources and increases risk of requiring mechanical ventilation and renal replacement therapy [15, 16]. In contrast, other studies demonstrated that the length of ED stay was not significantly associated with the outcome of critically ill medical patients [17, 18].

Time sensitive interventions often dictate outcomes in critically ill septic patients. Protocols have been actively implemented in the care of septic patients; however, the impact of emergency department length of stay (EDLOS) prior to ICU admission upon sepsis protocol compliance and outcomes needs further understanding. In this study, we investigated the relationship between EDLOS with outcomes and bundle compliance in patients presenting with severe sepsis or septic shock. The primary objective was to assess the impact of EDLOS on in-hospital mortality. The secondary objective was to assess the association of EDLOS with compliance with sepsis bundles and optimal resuscitation. Our study also examined whether code status affects the early management of critically ill septic patients.

## 2. Materials and Methods

### 2.1. Setting and Study Design

We present a retrospective observational study of critically ill septic patients admitted to the ICU from the ED at a single tertiary care hospital between January 2012 and December 2015. Patients included in the study were limited to those presenting with severe sepsis or septic shock and were admitted to the ICU from the ED. The definitions of severe sepsis and septic shock were adapted from the Surviving Sepsis Campaign of 2012 [19]. Sepsis is defined as the presence (probable or documented) of infection together with systemic manifestations of infection. Severe sepsis is defined as sepsis plus sepsis-induced organ dysfunction or tissue hypoperfusion, while septic shock is defined as sepsis-induced hypotension persisting despite adequate fluid resuscitation [19].

Demographics, vitals, laboratory data, microbiology data, medications, and hospital course were collected from the electronic medical records. Chart review and data collection were done by two critical care fellows who were supervised by a critical care attending physician. The study population was divided into 2 groups based on their EDLOS. The EDLOS is calculated as the time in hours from ED triage to the time that the patient was physically transferred to an ICU. Patients with EDLOS < 6 hrs were considered as “early admission,” while those admitted ≥ 6 hrs were considered “delayed admissions.” The decision to choose 6 hours as a cut-off time was based on the recommendation of the Canadian Association of Emergency Physicians (CAEP) on prolonged ED stay which was defined as EDLOS of more than 6 hours [8]. The assessment of quality of US ED care was also based on this cut-off time [9]. We also believe that 6 hours would suffice to carry out initial sepsis protocol. We excluded patients transferred to ED from another hospital's ED, long-term acute care (LTAC) facility, or postoperating room (OR) procedures. This study was approved by the St. Elizabeth's Medical Center Institutional Review Board, which waived the requirement for informed consent.

### 2.2. Sepsis Bundle, Resuscitation, and Compliance

The 3-hour bundle in our study was adapted from both previous (2008) and current (2012) sepsis bundles which include drawing an initial lactate level, obtaining blood cultures prior to antibiotic use, administering broad antibiotics, and providing IV hydration (30 cc/kg) when the patient is hypotensive or with elevated initial lactate (≥4 mmol/L). Compliance with the 3-hour bundle is defined as completion of all the four components.

The 2012 SSC 6-hour bundle has been streamlined with the recent ProCESS, ARISE, and ProMISe trials [20–22]. The measurement of central venous pressure (CVP) and mixed venous oxygen saturation (SVO_2_) was then taken out from the bundle. In that regard, we only included the use of vasopressors and remeasuring lactate if the initial is elevated and retermed this as “sepsis management beyond 3 hours” in our study. The indication for vasopressor use is hypotension (MAP < 65 mmHg) in the first 3 hours in the ED and persistence > 3 hours despite initial fluid resuscitation. Compliance was assessed on each of these protocol components.

Our study also assessed optimal resuscitation endpoints within 12 hours. Assessment of endpoints was limited to patients who were hypotensive (MAP < 65 mmHg) in the first 3 hours in the ED and persisted > 3 hours despite initial fluid resuscitation and/or elevated initial lactate (≥2.3 mmol/L) which were repeated within 12 hours. Lactate level of 2.3 mmol/L was the upper limit at our institution. Resuscitation endpoints were defined as a 20% drop of lactate level when the initial lactate level is elevated; achievement of optimal blood pressure was defined as 3 consecutive blood pressure readings of ≥MAP 65 mmHg on stable vasopressor use and urine output ≥ 0.5 cc/kg/hr. Patients were considered to be optimally resuscitated if 2 out of the 3 resuscitation endpoints were met. The study also assessed sepsis protocol compliance in relation to patient's code status (full code versus DNR/DNI).

### 2.3. Sample Size and Statistical Analysis

We powered this study to detect a mortality difference of 13% between the two groups (early versus delayed admission to ICU). Assuming that the mortality of delayed admission is 25%, 150 patients in each group provide 80% power, with alpha = 0.05, using a two-sided chi-square test. The categorical baseline characteristics as well as the outcomes of patients in the two groups were compared using the chi-square test. A logistic regression model was used to adjust for the potential confounders of SOFA, MAP, and lactate on mortality. The Wilcoxon rank-sum test was used to compare LOS, while *t*-test was used to compare all other continuous baseline characteristics. *p* value of <0.05 is considered significant.

## 3. Results

During the study period, 287 patients with severe sepsis and septic shock were admitted to the ICU from the ED. Of these, 137 (47.7%) were admitted early, while 150 (52.2%) had delayed ICU admission (Table 1). Age, sex, SOFA score, mean arterial pressure (MAP), hypotension (MAP < 65) on initial presentation, period of admission, and code status were not significantly different between the two groups (Table 1). However, statistical difference was observed with initial lactate level with patients admitted early having a slightly higher level (3.9 versus 3.2, *p* = 0.01). The number of patients with initial lactate level of ≥4 mmol/L was not significantly different between groups. Patients who stayed in the ED < 6 hours have a median LOS of 4.6 hours, while those with delayed ICU admission have a median EDLOS of 8.1 hours.

There was no significant in-hospital mortality difference between critically ill septic patients admitted early versus those with delayed admission in the ICU (22.63% versus 24.67%, *p* = 0.68; Table 2) (OR = 1.120; 95% CI: 0.649, 1.933; *p* = 0.69). The results were similar after adjusting for SOFA, MAP, and lactate (OR = 1.226; CI: 0.669, 2.247; *p* = 0.51). The median ICU length of stay was also not significantly different regardless of ICU transfer time using Wilcoxon's rank-sum test (2.2 versus 2.3 days, *p* = 0.65; Table 2). Similar results were observed when only those who survived were accounted for (2.2 versus 2.2 days, *p* = 0.09; Table 2).

The compliance with 3-hour sepsis protocol was also found to not be significantly different between two groups. There was a 50.36% compliance rate with early admission patients and 48.67% with patients with delayed admission (*p* = 0.77; Table 3). Patients admitted early in the ICU were more likely to have repeat lactate levels drawn if needed (63.48% versus 43.75%, *p* = 0.003; Table 3). However, we found no significant difference in compliance with vasopressor use between the two groups (59.21% versus 56.52%, *p* = 0.74; Table 3). Achievement of optimal resuscitation within 12 hours was observed in 63.64% of early admissions compared to 57.97% of delayed admission (*p* = 0.35; Table 3).

**Figure 1:**
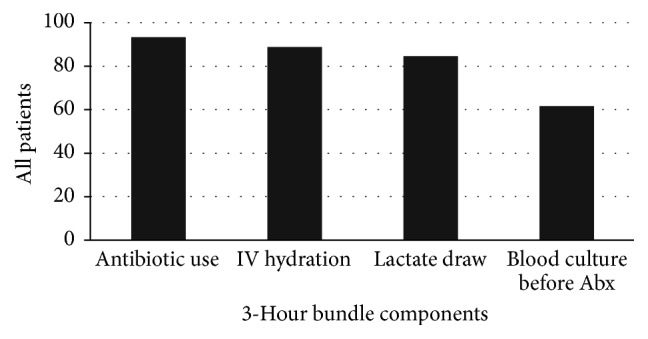
Compliance with individual components of 3-hour bundle.

Analysis of each component of the 3-hour bundle showed a high compliance rate with initial lactate measurement (84.66%), use of broad spectrum antibiotics (93.7%), and IV fluid hydration (88.8%). However, compliance with blood culture drawn prior to antibiotic was poor at 61.67% (Figure 1). The achievement of optimal urine output and blood pressure within 12 hours was 74.42% and 74.88%, respectively. Meanwhile the 20% reduction of an initially elevated lactate was only achieved in 44.91% of patients.

Subgroup analysis of full code patients showed similar trends in survival, sepsis bundle compliance, and optimal resuscitation in 12 hours (Table 4). There was no significant mortality difference between early and delayed ICU admissions despite having a full code status (17.82 versus 23.0, *p* = 0.35). Similarly, there was no significant difference in the compliance with 3-hour bundle, vasopressor, and optimal resuscitation after 12 hours. Patients with DRN/DNI status demonstrated a higher percentage of compliance with the 3-hour protocol compared to full code patients (Table 4). The overall compliance rates with the 3-hour bundle are similar between DNR/DNI patients admitted early and those admitted late in the ICU (36.11 versus 29.73, *p* = 0.56; Table 4). The previously noted higher compliance with lactate monitoring amongst early admitted patients was not observed in the DNR/DNI group (Table 4). The optimal resuscitation in 12 hours of DNR/DNI septic patients was not significantly different (60.61 versus 52.78, *p* = 0.51; Table 4).

Analysis on mortality alone showed that overall survival was greater in the full code group (*p* = 0.03; Table 5). Compliance with the 3-hour protocol was not significantly associated with improved survival (*p* = 0.86). Interestingly, achieving optimal resuscitation within 12 hours was associated with improved sepsis survival (*p* = 0.02; Table 5).

## 4. Discussion 

Our study did not find a significant mortality difference between critically ill septic ED patients admitted early (<6 hours) versus those with delayed admission (≥6 hour) to the intensive care unit. The results were similar after adjusting for SOFA, MAP, and lactate. The lengths of ICU stay were also similar between the two groups. Further analysis showed that the EDLOS did not significantly affect survival regardless of the patient's code status in the ED.

Our findings were consistent with previous studies that show no worsening of survival of critically patients despite their delayed ICU admissions [17, 18, 23]. Tilluckdharry et al. demonstrated that outcomes of patients admitted to MICU within 24 hours were not better than those who remained in the ED for a longer period of time [17]. However, this study included only 7% of patients with sepsis syndrome and shock. In a similar note, Saukkonen et al. showed that the length of ED stay was not associated with hospital mortality [18]. There are only few studies that investigated the ICU transfer time in the sepsis population. An observational study in Brazil showed that almost half of the delayed ICU admission with septic shock comes from the ED [24]. Another study suggests a 10% increase in mortality in each day of delayed ICU transfer of severely ill septic patients [25]. Our study used a particular cut-off time which is ≥6 hours to describe delayed ICU admission as recommended by the Canadian Association of Emergency Physicians on the definition of prolonged EDLOS. We believe that 6 hours of EDLOS is adequate to complete resuscitation protocols as directed by the SSC guidelines. Since there are no data at present to support the use of any particular time frame as an indicator of quality of care for septic patients in particular, we have initiated the use of a cut-off time to fill the void of knowledge. Our study demonstrated no difference in the outcomes of sepsis patients when they are dichotomously divided into those who stayed in the ED for <6 hours and those who stayed in the ED for ≥ 6 hours. It is possible that an effect may occur when different time interval is to be used.

The Surviving Sepsis Campaign focuses on compliance with sepsis bundle and its association with mortality. Levy et al. showed a 3–5% decrease in mortality with every 10% increase in compliance with sepsis protocol [4]. A sepsis campaign performance study published in 2010 showed a linear increase in the initial resuscitation bundle compliance (6-hour bundle) from 10.9% to 31.3% [3]. The compliance with initial lactate and blood culture draws, administration of antibiotics, and intravenous fluids resuscitation ranged from 67 to 79% [3]. Subsequent bundle performance analysis by Levy et al. in 2012 showed the compliance with all applicable elements of sepsis resuscitation bundle in the US (21.6%) versus Europe (18.4%) [26]. In our study, we demonstrated about 50% complete compliance with the 3-hour sepsis bundle which was patterned on the latest 2012 SSC guidelines. The use of antibiotics prior to blood culture has the highest compliance rate at 93.7%, while obtaining blood culture prior to antibiotic use was only 61.6%. The high rate of antibiotic utilization in the early part of hospitalization provides an outcome advantage in the care of septic patients. A study by Ferrer et al. showed a linear increase in the risk of mortality for every hour delay in antibiotic administration for patients with severe sepsis and septic shock [27]. Interestingly, a complete compliance with the 3-hour protocol in our study does not give any survival advantage to severely ill septic patients. Thus, the association of outcome with protocol compliance in this study should be generalized with caution.

The assessment of sepsis management beyond 3 hours has varying results. Higher compliance of lactate monitoring was observed in patients admitted to the ICU early. The higher rate of repeat lactate draw may not entirely be accounted to compliance as this may also reflect the habit of repeating blood works when patients reach the ICU. The higher level of initial lactate amongst the early admissions may have also prompted sequential draws. The length of ED to ICU transfer has not affected the use of vasopressors. We would reiterate, however, that the exact timing of vasopressor use in relation to onset hypotension was not specifically assessed in our study. A mere initiation of vasopressors when patients meet eligibility has defined compliance. This finding parallels a previous study which suggests that only marked delay (≥14 hours) brings about modest decrease in survival in patients with septic shock [28]. Lastly, analysis on overall mortality in our study showed no survival advantage on full compliance with 3-hour protocol during early part of resuscitation (Table 5).

The assessment of optimal resuscitation for septic patients, taken as a bundle, has not been investigated in the past. We suggest that lactate level, urine output, and blood pressure are key endpoint components of resuscitation in sepsis. A previous study showed that patients with higher lactate clearance of more than 10% within 6 hours of emergency department intervention have improved outcomes [29]. Our study used a cut-off of 20% decrease in lactate level in 12 hours. A lower resuscitation MAP threshold of 65 mmHg was also in conjunction with the study that showed that a targeted MAP of 65–70 did not show mortality difference with targeted MAP of 80–85 [30]. We have seen in our study that the timing of ICU admission did not significantly affect the achievement of optimal resuscitation within 12 hours. When individual endpoints were compared, achievement of optimal BP and UO was observed in about 74%, while the drop on lactate was poorly achieved in 45%. Subgroup analysis showed that patients who are optimally resuscitated within 12 hours have lower mortality compared to those who did not achieve optimal resuscitation (18.4% versus 31.3%). Future studies are recommended to see if optimal resuscitation bundle is a better marker for sepsis prognosis compared to protocol compliance.

Our study has also examined whether code status affects the early management of critically ill septic patients. A previous study showed that about half of septic patients with DNR/DNI status survived to hospital discharge [31]. DNR patients also received invasive measures at a rate similar to that of patients without DNR status [31]. Our subgroup analysis showed that compliance with 3-hour protocol, vasopressor use, and achievement of optimal resuscitation in each respective code status were not affected by the timing of ED to ICU transfer. However, a peculiar finding was noted where patients with DNR/DNI status have a higher percentage of compliance with the 3-hour protocol in general compared to the full code group (Table 4). Thus, the presence of DNR/DNI status does not preclude the initiation of early resuscitation in our institution. It is still debatable whether to include patients with DNR/DNI status in the assessment of performance metrics of sepsis management.

*Limitations*. There are limitations to be considered in this study. This is a retrospective observation study from a single institution collected over the span of 4 years. Observational studies are susceptible to selection bias, which can interfere with results. Our study was powered to detect a 13% absolute difference in mortality given that 150 patients are included in each group. The study is underpowered to detect predetermined difference since the early admission group has only 137 patients. The definition of prolonged EDLOS and access block may vary. Prolonged ED visit has variously been defined as >4 hours in the United Kingdom, >6 hours in Canada/US, and >8 hours in Australia. We used the CAEP recommendation based on their position statement on emergency overcrowding in 2009. Future studies should explore other cut-off times. We also believe that a subtle evolution of illness in the ED may occur and could have somehow dictated the timing of ICU admission. Likewise, patient may also have improved dramatically in the ER where ICU admission was not necessary. It is also difficult to determine whether the delayed decision to admit to ICU was due to poor triage versus unavailability of ICU beds. Further studies on the risk factors leading to delayed admission are recommended. Other hospitals vary in emergency response team and may have a dedicated sepsis code team, so our results must be generalized with caution. The subgroup analysis may not have the power to detect difference in sepsis outcomes, protocol compliance, and resuscitation. This study did not individually identify the sources of sepsis, those who required invasive ventilation, or dialysis. These factors may impact mortality despite identical severity score amongst patient groups. Source control and steroids use as part of sepsis management were not included in the analysis of the study.

## 5. Conclusion 

Sepsis protocols are currently in wide use and have promoted the standardization of care for ED septic patients. We found that clinical outcomes were not significantly different between early and delayed ICU admissions. Additionally, EDLOS did not significantly affect compliance with the sepsis protocol with the exception of adherence to repeat lactate testing.

## Figures and Tables

**Table 1:** Demographics and baseline characteristics for early and delayed ICU admission (*n* = 287).

	Early admission <6 hours (*n* = 137)	Delayed admission ≥6 hours (*n* = 150)	*p* value
Age (mean, SD)	73.0 (13.6)	72.4 (16.0)	0.73
Sex (% male)	53.3	48.0	0.37
Admission per calendar quarter (%)			0.38
Q1 (January–March)	25.55	34.00	
Q2 (April–June)	36.50	29.33	
Q3 (July–September)	18.98	20.00	
Q4 (October–December)	18.98	16.67	
SOFA score (mean, SD)	7.2 (3.2)	7.0 (3.3)	0.56
MAP (mean, SD)	70.1 (19.5)	72.0 (18.6)	0.40
Initial lactate (mean, SD)	3.9 (2.6)	3.2 (2.2)	0.01
Patient with initial lactate ≥ 4 (%)	39.4	30.0	0.09
Hypotension (MAP < 65) on initial presentation	49.64	40.67	0.13
Code status			0.75
Full	73.72	75.33	
DNR/DNI	26.28	24.67	
Median ED time (hours) [25th, 75th percentile]	4.6 [3.8, 5.3]	8.1 [7.1, 10.8]	

MAP, mean arterial pressure; SOFA, Sequential Organ Failure Assessment; DNR, do not resuscitate; DNI, do not intubate.

**Table 2:** Survival and ICU length of stay.

	Early admission <6 hours	Delayed admission ≥6 hours	*p* value
*All patients*	(*n* = 137)	(*n* = 150)	
Mortality	22.63 (15.62, 29.63)	24.67 (17.77, 31.57)	0.68
Median ICU length of stay of all patients (days) [25th, 75th percentile]	2.2 [1.2, 3.5]	2.3 [1.3, 4.2]	0.65
*All survivors*	(*n* = 106)	(*n* = 113)	
Median ICU length of stay of all survivors (days) [25th, 75th percentile]	2.2 [1.4, 3.5]	2.2 [1.4, 4.2]	0.99

**Table 3:** Compliance with sepsis bundles and optimal resuscitation within 12 hours.

	Early admission <6 hours (*n* = 137)	Delayed admission ≥6 hours (*n* = 150)	*p* value
*3-Hour resuscitation bundle*			
Compliance with 3-hour protocol	50.36 (41.99, 58.74)	48.67 (40.67, 56.67)	0.77
*Management beyond 3 hours*			
Compliance with lactate monitoring	63.48 (54.68, 72.28)	43.75 (34.56, 52.94)	0.003
Compliance with vasopressor use	59.21 (48.16, 70.26)	56.52 (44.82, 68.22)	0.74
*Resuscitation outcome*			
Optimal resuscitation within 12 hours	63.64 (55.07, 72.21)	57.97 (49.74, 66.21)	0.35

**Table 4:** Compliance with sepsis bundles and optimal resuscitation within 12 hours comparing code status (full code versus DNR/DNI).

	Full code (*n* = 214)			DNR/ DNI (*n* = 73)		
	Early admission (*n* = 101)	Delayed admission (*n* = 113)	*p* value	Early admission (*n* = 36)	Delayed admission (*n* = 37)	*p* value
Mortality	17.82	23.01	0.35	36.11	29.73	0.56
Compliance with 3-hour protocol	46.53	44.25	0.74	61.11	62.16	0.93
Compliance with lactate monitoring	63.41	43.21	0.001	63.64	45.16	0.14
Compliance with vasopressor use	58.18	56.86	0.89	61.90	55.56	0.69
Optimal resuscitation within 12 hours	64.77	59.80	0.48	60.61	52.78	0.51

**Table 5:** Subgroup analysis of mortality.

	Survivors	*p* value
Code status		0.03
Full code (*n* = 214) (%)	79.44	
DNR/DNI (*n* = 73) (%)	67.12	
Compliance with 3-hour protocol		0.86
Compliant (*n* = 142) (%)	76.76	
Noncompliant (*N* = 145) (%)	75.86	
Resuscitation endpoints within 12 hours		0.02
Optimal resuscitation (*n* = 157) (%)	81.53	
Suboptimal resuscitation (*n* = 102) (%)	68.63	
